# Exogenous R-Spondin1 Induces Precocious Telogen-to-Anagen Transition in Mouse Hair Follicles

**DOI:** 10.3390/ijms17040582

**Published:** 2016-04-20

**Authors:** Na Li, Shu Liu, Hui-Shan Zhang, Zhi-Li Deng, Hua-Shan Zhao, Qian Zhao, Xiao-Hua Lei, Li-Na Ning, Yu-Jing Cao, Hai-Bin Wang, Shuang Liu, En-Kui Duan

**Affiliations:** 1 State Key Laboratory of Stem Cell and Reproductive Biology, Institute of Zoology, Chinese Academy of Sciences, Beijing 100101, China; linawhere@163.com (N.L.); treetreew@gmail.com (S.L.); zhanghs@ioz.ac.cn (Hui.-S.Z.); dengzhili1024@163.com (Z.-L.D.); zhaohuashan@gmail.com (Hua.-S.Z.); leixh@ioz.ac.cn (X.-H.L.); ningln@ioz.ac.cn (L.-N.N.); caoyj@ioz.ac.cn (Y.-J.C.); hbwang@ioz.ac.cn (H.-B.W.); 2 University of Chinese Academy of Sciences, Beijing 100049, China; 3 State Key Laboratory of Agrobiotechnology, College of Biological Sciences, China Agricultural University, Beijing 100193, China; bingxia0923@163.com

**Keywords:** R-spondin, Wnt/β-catenin, hair follicle stem cells, hair cycle

## Abstract

R-spondin proteins are novel Wnt/β-catenin agonists, which signal through their receptors leucine-rich repeat-containing G-protein coupled receptor (LGR) 4/5/6 and substantially enhance Wnt/β-catenin activity. R-spondins are reported to function in embryonic development. They also play important roles in stem cell functions in adult tissues, such as the intestine and mammary glands, which largely rely on Wnt/β-catenin signaling. However, in the skin epithelium and hair follicles, the information about R-spondins is deficient, although the expressions and functions of their receptors, LGR4/5/6, have already been studied in detail. In the present study, highly-enriched expression of the R-spondin family genes (*Rspo1/2/3/4*) in the hair follicle dermal papilla is revealed. Expression of *Rspo1* in the dermal papilla is specifically and prominently upregulated before anagen entry, and exogenous recombinant R-spondin1 protein injection in mid-telogen leads to precocious anagen entry. Moreover, R-spondin1 activates Wnt/β-catenin signaling in cultured bulge stem cells *in vitro*, changing their fate determination without altering the cell proliferation. Our pioneering study uncovers a role of R-spondin1 in the activation of cultured hair follicle stem cells and the regulation of hair cycle progression, shedding new light on the governance of Wnt/β-catenin signaling in skin biology and providing helpful clues for future treatment of hair follicle disorders.

## 1. Introduction

The Wnt/β-catenin signaling is accepted as the dominant player regulating multiple aspects of skin biology, such as the morphogenesis and patterning of skin epithelium and its appendages, the self-renewal and differentiation of resident stem cells in skin, as well as the functions of numerous differentiated skin cells [1]. Hair follicles are important epidermal appendages which go through repeated cycles of catagen (degeneration), telogen (rest), and anagen (growth) during postnatal life. The hair cycle is controlled by extensive interactions between the hair follicle epithelial cells and the local dermal cells, especially those in the dermal papilla (DP), an organized structure at the bottom of a hair follicle which is comprised of specialized dermal mesenchymal cells [2,3]. In these complex epithelial-mesenchymal interactions and the entire hair cycle regulation, the Wnt/β-catenin pathway plays diverse and essential roles at different aspects, for example, the transition from telogen to anagen, and the lineage decisions during hair follicle differentiation.

The newly-discovered R-spondin1-4 is a small family of vertebrate-specific secreted growth factors that strongly enhance Wnt/β-catenin activity by collaborating with their receptor LGR4/5/6 [4,5,6,7,8] and the transmembrane E3 ligases ZNRF3/RNF43 [9]. Increasing studies have underlined the role of R-spondin proteins in embryonic development, stem cell biology and human diseases. In highly-regenerative epithelial tissues such as the intestine and the mammary gland, where the canonical Wnt/β-catenin pathway has a well-established position, R-spondins are found to play especially critical roles, not only in the tissue development, but also in adult stem cell functions [5,10,11,12].

Several studies have revealed the implication of R-spondins in the skin. In particular, R-spondin2 is reported to be associated with hair coat characteristics in dogs [13]. Interestingly, lineage tracing studies in mice demonstrated the importance of R-spondin receptors LGR proteins in the skin epithelium: LGR6 marks the primitive embryonic hair placode epidermal stem cells that generate the hair follicle and sebaceous gland [14], and LGR5 marks the actively cycling adult hair follicle stem cells (HFSCs) that are responsible for the cyclic regeneration of the hair follicles [15]. Importantly, as mentioned above, the cyclic activation of HFSCs at the telogen-to-anagen transition is Wnt/β-catenin-dependent. Previous microarray data indicated an upregulation of R-spondin1 and R-spondin2 expression in DP before anagen [16]. Therefore, it is of great possibility that R-spondins play a role in HFSC activation and anagen initiation during the hair cycle progression.

Herein, we examined the spatiotemporal expressions of R-spondins in the telogen hair follicles and revealed especially the role of R-spondin1 in the induction of anagen and the regulation of HFSC activation. R-spondin family genes (*Rspo1/2/3/4*) were highly expressed in the hair follicle dermal papilla, and the expression of *Rspo1* prominently was upregulated before anagen entry. *In vivo*, the precocious anagen entry happened because of the exogenous recombinant R-spondin1 protein injection. *In vitro*, R-spondin1 activated Wnt/β-catenin signaling in cultured bulge stem cells, changing their fate determination without altering the cell proliferation. In conclusion, our study demonstrated an unrecognized role of R-spondin1 mediating between the DP and the hair follicle to initiate regenerative hair cycling.

## 2. Results

### 2.1. R-Spondin1-4 Expression Is Enriched in the Telogen Hair Follicle Dermal Papilla, and R-Spondin1 Expression Is Upregulated before Anagen Entry

**Figure 1:**
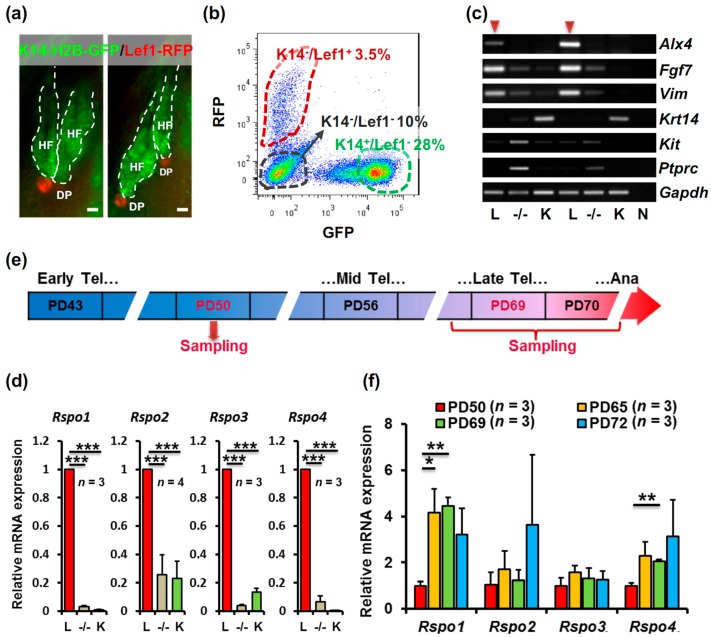
Expression of R-spondin genes in the mouse dorsal skin. (**a**) Hair follicles in the back skin of a K14-H2B-GFP/Lef1-RFP mouse; (**b**) isolation of different cell populations from the dorsal skin of K14-H2B-GFP/Lef1-RFP mice using FACS; (**c**) RT-PCR results showing the expression of different marker genes in the K14^−^/Lef1^+^ (L), K14^−^/Lef1^−^ (−/−) and K14^+^/Lef1^−^ (K) cell populations from two independent mice. H_2_O was used instead of cDNA as a negative control (N) and *Gapdh* was used as an internal control. The arrows indicate the track of K14^−^/Lef1^+^ (L) group; (**d**) qPCR results showing the relative expression of the four R-spondin genes in the K14^−^/Lef1^+^ (L), K14^−^/Lef1^−^ (−/−) and K14^+^/Lef1^−^ (K) cell populations; (**e**) sampling schedule for the FACS assay; and (**f**) relative expression of R-spondin genes in the DP in different periods of telogen. HF, hair follicle; DP, dermal papilla; Tel, telogen; Ana, anagen; PD, postnatal day. Bar = 50 μm. * *p* < 0.05; ** *p* < 0.01; *** *p* < 0.001.

To determine whether R-spondins function in anagen initiation, we first located the expression of R-spondin genes in different cell populations in the telogen mouse skin. K14-H2B-GFP/Lef1-RFP double transgenic mice were used to isolate epidermal cells, dermal fibroblasts and DP cells [17]. In telogen mouse skin, the K14^+^ epidermal cells from the hair follicle outer root sheath showed nuclear green fluorescent protein (GFP) fluorescence and the Lef1^+^ DP was specifically labeled with red fluorescent protein (RFP) fluorescence (Figure 1a). By fluorescence activated cell sorting (FACS), three populations of cells were sorted (Figure 1b): the K14^−^/Lef1^+^ cells which contained the DP cells and a few melanocytes, the K14^+^/Lef1^−^ epidermal cells and the K14^−^/Lef1^−^ cells which were mostly dermal fibroblasts and some other cells such as immune cells. In order to evaluate the purity of DP cells in the K14^−^/Lef1^+^ population, markers for DP cells, melanocytes and immune cells were assessed. Immunostaining results indicated that the K14^−^/Lef1^+^ population contained only a very small portion of CD45^+^ immune cells and CD117^+^ melanocytes (Figure S1, upper left panels), whereas the K14^−^/Lef1^−^ population contained more CD117^+^ melanocytes (Figure S1, lower panels). Besides, the K14^−^/Lef1^+^ cells exhibited evident alkaline phosphatase (AP) activity (Figure S1, upper right panel), which is a characteristic of DP cells. Moreover, RT-PCR was performed to examine different markers in sorted populations from two individual mice. As shown in Figure 1c, in the case of twice repeat, K14^−^/Lef1^+^ population displayed strongest expression of DP markers *Alx4*, *Fgf7*, and *Vim*, and no or very weak expression of epidermal gene *Krt14*, melanocyte marker *Kit* (CD117 gene) and immune cell marker *Ptprc* (CD45 gene). Therefore, the vast majority of K14^−^/Lef1^+^ cells we sorted were DP cells.

After the sorted skin cell populations were identified, R-spondin1-4 expression in these populations was compared. Interestingly, all of the four R-spondin genes were highly enriched in DP cells (Figure 1d). To be specific, for the K14^−^/Lef1^+^ cells, Δ*C*_t*Rspo1*_ (C_t*Rspo1*_ − C_t*Gapdh*_) was 3.33 ± 0.56; ΔC_t*Rspo2*_ was 6.81 ± 1.47; Δ*C*_t*Rspo3*_ was 8.82 ± 0.80; Δ*C*_t*Rspo4*_ was 4.63 ± 0.85. It is well-known that DP signals are important in regulating HFSC activation to initiate anagen. To determinate the correlation of R-spondin genes in DP cells and the hair cycle, we next investigated the dynamic expression changes of these genes during telogen. After cell populations sorted as indicated in Figure 1e, mRNA expressions of R-spondin genes in DP at early-to-mid telogen (postnatal day 50, PD50), late telogen (PD65–PD69) and early anagen (PD72) were compared. The results exhibited, during the telogen phase, while *Rspo2* and *Rspo3* mRNA levels were relatively stable, *Rspo1* and *Rspo4* mRNA levels showed significant upregulation in late telogen (Figure 1f), specifically, Δ*C*_t*Rspo1*_ was 5.11 ± 0.26 on PD50, 2.95 ± 0.42 on PD65, 2.75 ± 0.13 on PD69, 3.43 ± 0.55 on PD72; Δ*C*_t*Rspo4*_ was 6.01 ± 0.16 on PD50, 4.91 ± 0.50 on PD65, 4.99 ± 0.06 on PD69, 4.78 ± 0.82 on PD72. *Rspo1* mRNA level on PD65 and PD69 was four times of that on PD50 and it exhibited a slight down-trend in PD72. However, due to unsynchronized anagen entry, PD72 R-spondin mRNA levels showed considerable variation. Interestingly, we observed similar expression pattern of several Wnt related genes in DP, suggesting a correlation between Wnt activation and *Rspo1*/*4* expression in DP (Figure S2).

### 2.2. R-Spondin1 Injection Leads to Precocious Anagen Entry

**Figure 2:**
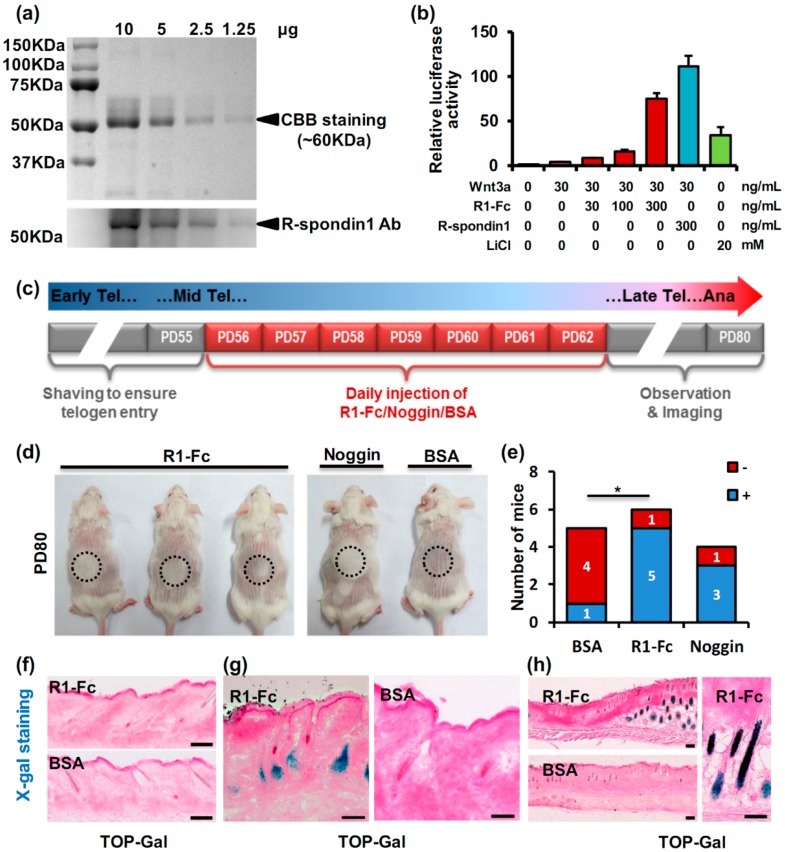
Exogenous R-spondin1 injection leads to precocious anagen entry. (**a**) Coomassie blue staining and immunoblotting of R-spondin1-Fc protein purified from the R-spondin1-293T cells; (**b**) TCF luciferase assay showing the bioactivity of the R-spondin1-Fc protein; (**c**) schedule for R-spondin1 injection assay; (**d**) pictures of mice injected with R-spondin1-Fc, Noggin or BSA (negative control) on PD80. Dotted circles show the injection site; (**e**) statistical analysis of the protein injection assay. Mice with precocious hair regrowth was regarded as + and the opposite as −; (**f**) X-gal staining in dorsal skin of TOP-Gal mice on PD55; (**g**) X-gal staining of dorsal skin of TOP-Gal mice injected with R-spondin1-Fc or BSA one week after the initial injection; an (**h**) X-gal staining of dorsal skin of TOP-Gal mice injected with R-spondin1-Fc or BSA about three weeks after the initial injection. CBB, Coomassie blue; R1-Fc, R-spondin1-Fc; PD, postnatal day; BSA, bovine serum albumin; Tel, telogen; Ana, anagen. Bar = 100 μm in (**f**,**g**). * *p* < 0.05.

The upregulation of *Rspo1* expression in DP during late telogen suggested a possible role of this gene in anagen induction. To investigate this possibility, recombinant R-spondin1-Fc protein was produced by modified 293T cells, purified from the supernatant and injected intradermally into the mid-telogen mice skin. The production of the R-spondin1-Fc protein was verified by Coomassie Brilliant Blue (CBB) staining and anti-R-spondin1 antibody immunoblotting. The bands for R-spondin1-Fc protein were detected around 60 KDa (Figure 2a), which was consistent with previously reported results [18,19]. Bioactivity of the R-spondin1-Fc protein was examined with a T-cell factor (TCF)-luciferase assay in 293T cells. As shown in Figure 2b, compared with the positive control LiCl, R-spondin1-Fc protein highly stimulated TCF-luciferase activity in a dose-dependent manner in combination with a consistent dose of Wnt3a. The comparison of R-spondin1-Fc protein with commercial recombinant human R-spondin1 protein indicated a slightly lower but acceptable activity of our R-spondin1-Fc protein (Figure 2b). Therefore, given the amount of protein needed for *in vivo* injection and the cost, we used R-spondin1-Fc protein rather than commercial recombinant R-spondin1.

To investigate the role of R-spondin1 in the telogen-to-anagen transition, the R-spondin1-Fc protein we produced was injected intradermally into the dorsal skin of mid-telogen (PD56) mice for one week according to a schedule shown in Figure 2c. Noggin was injected as a positive control [20,21], and a bovine serum albumin (BSA) solution was also injected as the negative control. New hairs could be observed at the injection site in R-spondin1-Fc and Noggin mice as early as PD75 and became quite evident on PD80 (Figure 2d), whereas BSA-injected control mice showed no obvious hair growth at that time point. A statistical analysis of hair growth was performed to eliminate the effect of unsynchronized anagen entry among individual mice. As indicated in Figure 2e, there were significant differences between the negative control group and the R-spondin1-Fc group.

The advanced appearance of new hairs at the injection site indicated a precocious anagen entry, suggesting that R-spondin1 promoted the induction of anagen in hair follicles. To study the precocious anagen entry in detail, we also performed the injection experiments on TOP-Gal mice, in which the activation of Wnt/β-catenin could be monitored through the specific X-gal staining. There were all-telogen hair follicles and no X-gal staining in TOP-Gal mice on PD55 (the time before injection) (Figure 2f). One week after the initial injection, early anagen hair follicles were observed in the R-spondin1-Fc-injected site with obvious X-gal staining, indicating the activation of the Wnt/β-catenin pathway, whereas control mice still had telogen hair follicles and negligible X-gal staining (Figure 2g; Figure S3). Some hair follicles with X-gal staining in the BSA injection site were detected around PD76, as shown in Figure S4. Later, full anagen hair follicles with intense X-gal staining were found in the R-spondin1-Fc-injection site, in drastic contrast to the non-injection site and the BSA injection site (Figure 2h).

### 2.3. R-Spondin1 Activates Wnt/β-Catenin Pathway in Bulge Stem Cells and Regulate HFSC Fate Determination in Vitro

**Figure 3:**
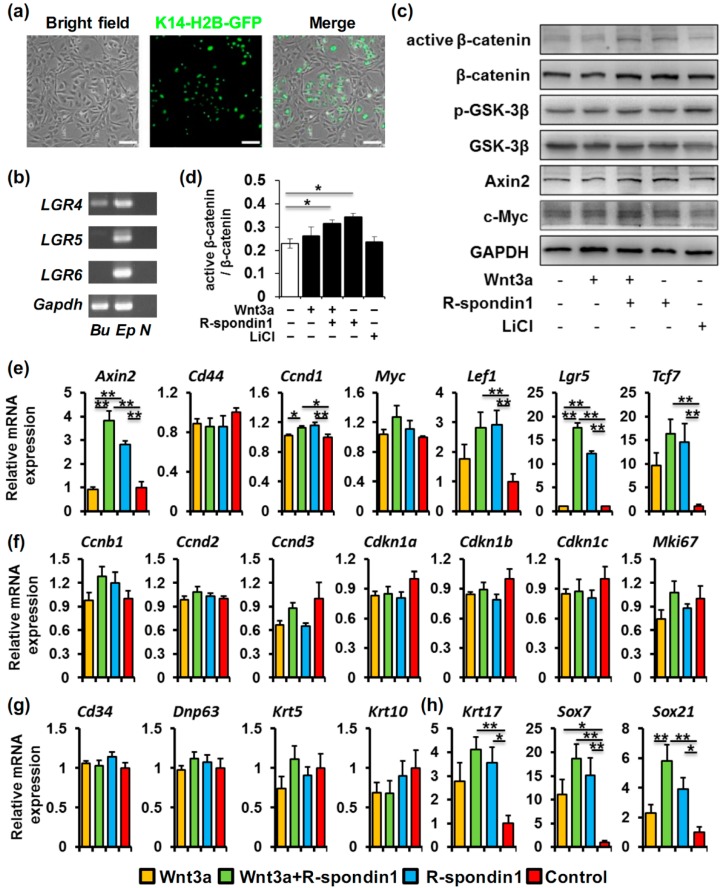
R-spondin1 activates Wnt/β-catenin signaling in bulge stem cells *in vitro*. (**a**) Morphology and green fluorescence of K14-H2B-GFP bulge stem cells in culture. The non-GFP cells were NIH-3T3 feeder cells; (**b**) RT-PCR showing the expression of *Lgr4/5/6* in the cultured bulge stem cells (Bu). Mouse epidermal cells (Ep) were used as a positive control and H_2_O was used as a negative control (N); (**c**) Western blotting showing the activation of Wnt/β-catenin in bulge stem cells treated with LiCl, Wnt3a or/and R-spondin1. GAPDH was used as a loading control; (**d**) densitometric analysis of active β-catenin/β-catenin in Figure 3c. Three independent experiments were performed for quantification; (**e**–**h**) qPCR results showing relative expression of Wnt/β-catenin target genes (**e**); cell cycle-related genes (**f**); genes associated with HFSC differentiation (**g**) and fate determination (**h**) in bulge stem cells treated with Wnt3a or/and R-spondin1. Bar = 100 μm. * *p* < 0.05; ** *p* < 0.01.

One inevitable event of the anagen entry is the HFSC activation. Since R-spondin1 injection can trigger the earlier anagen entry in the hair cycle, the effect of R-spondin1 on HFSCs was further investigated *in vitro*. The hair follicle bulge stem cells we used were originally derived from the K14-H2B-GFP mice based on GFP and CD34 expression as previously described [22]. The cells exhibited strong nuclear GFP fluorescence due to the K14 promoter activity whereas the feeder cells showed no fluorescence (Figure 3a). We first assessed the expression of R-spondin1 receptors in these cells. As shown in Figure 3b, *Lgr4* was detected in the cultured bulge stem cells by RT-PCR, whereas *Lgr5* and *Lgr6* were absent.

Given the expression of R-spondin receptor LGR4 in the bulge stem cells, these cells were starved and treated with Wnt3a and/or R-spondin1, and then the activation of Wnt/β-catenin signaling was studied. Western blotting results showed an obvious activation of β-catenin after the addition of either Wnt3a+R-spondin1 or R-spondin1 alone (Figure 3c, Lane 3 and 4; Figure 3d), whereas Wnt3a treatment alone showed little effect (Figure 3c, Lane 2; Figure 3d). The expressions of the downstream effectors, the phosphorylation of GSK-3β and Axin2, a Wnt target, were slightly enhanced by the treatments of Wnt3a + R-spondin1 or R-spondin1 alone (Figure 3c). However, the change of the expression of the Wnt target c-Myc, which is an important transcription factor implicated in keratinocyte proliferation, is not evident (Figure 3c). Furthermore, expression of other Wnt target genes was assessed after 24 h of treatment using qPCR (Figure 3e). Consistent with the Western blotting results, *Axin2* expression was elevated by the treatments of R-spondin1 or Wnt3a + R-spondin1 whereas *Myc* expression was not altered. Similarly, *Lef1* and *Tcf7* expression was also upregulated. Interestingly, *Lgr5* upregulation was prominent by R-spondin1 supplementation whereas Wnt3a alone showed no effect at all. We also observed a very subtle augment in Cyclin D1 (*Ccnd1*) mRNA level in R-spondin1 and Wnt3a + R-spondin1 groups, suggesting possible regulation of HFSC proliferation by R-spondin1.

To further elucidate the possible effect of R-spondin1 on the bulge stem cells *in vitro*, we assessed the expression of genes that are associated with cell proliferation, differentiation, and hair follicle fate determination. Many of these genes are also reported to be Wnt/β-catenin targets. We first examined genes involved in cell cycle regulation, quiescence maintenance, and cell proliferation, including cyclins (*Ccnb1/2/3*), cyclin-dependent kinase inhibitors (*Cdkn1a/1b/1c*) and *Mki67* (Figure 3f). To our surprise, none of the analyzed genes showed any change, suggesting no or little alteration in bulge stem cell proliferation after R-spondin1 treatment. This was re-verified by the MTS cell proliferation assay which showed no remarkable difference between groups after 24 h of treatment (Figure S5). The activation of HFSCs could also be attributed to cell differentiation or change in fate determination. Therefore, we next examined the expression of genes involved in HFSC differentiation (Figure 3g) and hair follicle fate determination (Figure 3h). According to our qPCR results, there was no significant change in the expression of HFSC markers *Cd34*, *Trp63*, *Krt5*, and the early differentiated keratinocyte marker *Krt10*, suggesting no obvious sign of bulge stem cell differentiation after 24 h of R-spondin1 treatment. However, the hair follicle fate-related genes *Krt17*, *Sox7*, and *Sox21* already showed substantial upregulation after the treatment with R-spondin1 or Wnt3a+R-spondin1, whereas Wnt3a treatment alone had a much weaker impact.

Since the R-spondin1 treatment alone showed a considerable effect in the assays above, we speculate that there might already be considerable endogenous Wnt ligands in the culture system. RT-PCR assay revealed the expression of many Wnt ligands, such as *Wnt4* and *Wnt10a* in the bulge stem cells themselves and *Wnt3a*, *Wnt5b*, *Wnt9b*, and *Wnt10b* in the feeder 3T3 cells (Figure S6).

## 3. Discussion

Although R-spondin proteins have been demonstrated to function in multiple tissues, the specificity and location of R-spondin synthesis have not been well-elucidated. More importantly, in the skin hair follicles, information about R-spondins is relatively lacking, although the expressions and functions of their receptors, LGR4/5/6, are studied in detail. Herein, we demonstrated the enrichment of R-spondins expression in hair follicle DP cells, the effect of R-spondin1 protein on anagen induction and the impact of R-spondin1 on HFSCs *in vitro*, shedding light on the questions mentioned above.

The expression of R-spondins in the skin was occasionally reported. In an earlier study, it was mentioned that expression of R-spondin1 was seen in patches of the developing mouse dermis at E12.5 and then restricted in the DP since E18.5 [23]. *Rspo3* and *Rspo4* mRNA was found restricted in the mouse nail field mesenchyme at E14.5 [24]. Studies in humans also pointed out that expression of *Rspo1*, *Rspo2*, and *Rspo4* was mainly found in dermal fibroblasts, but not in keratinocytes [25,26]. The mesenchymal distribution of R-spondin genes is also found in many other embryonic tissues such as the mammary gland (*Rspo1*) [27], the branch arch (*Rspo2*) [28], the teeth (*Rspo2*) [29], the lung (*Rspo2*) [30], *etc.*, and proved to regulate the morphogenesis of these tissues [28,30]. In the present study, definitive enrichment of the expression of all four R-spondin genes in the DP was revealed, indicating the hair follicle mesenchyme as one site for R-spondin protein synthesis in the skin, which is in accordance with the studies mentioned above. Although all of the four R-spondin genes are enriched in the telogen DP, only *Rspo1* and *Rspo4* expression exhibited significant upregulation right before anagen entry when compared with early-mid telogen. The increase in *Rspo1* expression is consistent with the previous microarray data in which DP gene expression on late telogen (PD69) and early telogen (PD43) were compared [16]. Interestingly, we observed similar expression pattern of several Wnt related genes in DP, suggesting a correlation between Wnt activation and *Rspo1*/*4* expression in DP. Furthermore, the role of R-spondin4 in regulation of the hair cycle need to be further studied.

During telogen, the hair follicle is kept in a resting status mainly by bone morphogenetic protein (BMP) signaling, and the antagonistic interplay between BMP, Wnt/β-catenin, and some other signaling, such as TGF-β, regulates the choice for HFSC activation and anagen entry [1]. Although it is agreed that, physiologically, Wnt/β-catenin activity in both the DP and the hair follicle triggers the transition to anagen [31], the exact detail of this signaling activity is still under debate, and the source and precise Wnt ligand(s) involved in this process remain unknown [1]. The conventional view holds that there is no or very low Wnt/β-catenin activity throughout telogen until the telogen-anagen transition, because Wnt ligand expression (Wnt10a, Wnt10b, and Wnt5a) could not be detected at this time point [32]. However, another view is that Wnt/β-catenin activity is present in the competent telogen (usually the late period of telogen with low BMP signaling level [33,34]) and the increase in the relative Wnt/β-catenin signaling level led to surpass a threshold and initiate anagen entry. Given the ability of R-spondin proteins to greatly potentiate Wnt signaling, our observation that R-spondin1 expression in DP largely increase in late telogen prompted us to speculate that R-spondin1 might be involved in the increase of the Wnt/β-catenin signaling level during the anagen induction. Expectedly, our injection experiments proved the capacity of exogenous R-spondin1 to initiate anagen. More importantly, the precocious anagen entry induced by R-spondin1 injection alone in mid-telogen (PD56–PD62) suggests that there is already a certain level of Wnt/β-catenin activity during this phase, because R-spondin1 can only enhance Wnt/β-catenin activity in the presence of other Wnt ligand(s). The timing of our R-spondin1-induced precocious hair growth is in conformity with TGF-β2-induced hair growth reported by Oshimori *et al.* [22]. Nevertheless, it is still worth further investigation whether the DP-derived R-spondin1 in late telogen triggered the anagen initiation in normal hair cycle progression. DP-specific *Rspo1* knockout would help address this question.

We further studied the effect of R-spondin1 on bulge stem cells *in vitro* to help find possible mechanism(s) through which exogenous R-spondin1 injection promote anagen entry. It is reported that LGR5 is strongly expressed in the lower portion of the CD34^+^ bulge stem cells in telogen [15], but the cultured bulge stem cell line we used in the *in vitro* assays which was originally derived from telogen mouse back skin based on CD34 expression [16] did not show LGR5 expression. It is possible that the LGR5 expression is lost during the long-time amplification *in vitro*. Nevertheless, these bulge stem cells express another R-spondin1 receptor, LGR4, which, we believe, makes them responsive to R-spondin1. In our *in vitro* study, R-spondin1 supplementation alone exhibits a considerable effect on the enhancement of Wnt/β-catenin activity, indicating the existence of endogenous Wnt ligands. Indeed, we detected there were the expression of several Wnt ligands, either in bulge stem cells or in the feeder cells, such as Wnt3a, Wnt4, Wnt5, and Wnt10a. Therefore, we pondered R-spondin1 might exaggerate the Wnt/β-catenin activity in the presence of some of these Wnt ligands.

Several of the Wnt target genes that were upregulated by R-spondin1 (or together with Wnt3a) in the bulge stem cells *in vitro*, such as *Axin2* and *Lef1*, are reported to be associated with HFSC activation *in vivo* upon telogen-to-anagen transition [35]. LGR5, which marks proliferative, but not quiescent, stem cells in the gastrointestinal tract and the hair follicle is also a Wnt target, *per se*. Intriguingly, as mentioned above, LGR5 was initially absent in the bulge stem cells *in vitro*, but it showed a drastic upregulation with R-spondin1 treatment (but not with Wnt3a alone), implying the potential importance of R-spondin1 in HFSC activation. We did not detect evident changes in the expressions of genes associated with cell proliferation (*Ccnb1*, *Ccnd1/2/3*, *Mki67*, *etc.*), but substantial change in HFSC fate related genes which are also Wnt/β-catenin targets (*Krt17*, *Sox7*, and *Sox21*) occurred. Based on these data, it is speculated that R-spondin1-mediated Wnt/β-catenin activation does not directly regulate HFSC proliferation *in vitro*, but possibly control HFSC fate determination and in turn initiates HFSC activation.

In conclusion, we herein reveal highly enriched expression of R-spondin genes in the hair follicle DP in the mouse back skin and a specific increase in *Rspo1* expression level upon telogen-to-anagen transition. Exogenous R-spondin1 protein injection in the mid-telogen induces precocious anagen entry in the hair follicle. Moreover, R-spondin1 protein activates Wnt/β-catenin in HFSCs *in vitro*, possibly affecting HFSC fate determination, but does not alter HFSC proliferation. Given the central role of Wnt/β-catenin in the homeostasis and regeneration of the skin and the hair follicles, our study provides helpful clues for the future treatment of disorders such as alopecia.

## 4. Materials and Methods

### 4.1. Mice

The K14-H2B-GFP/Lef1-RFP double transgenic mice were a kind gift from Elaine Fuchs’ lab and were originally derived as previously described [17]. The TOP-Gal mice were purchased from the RIKEN Biosource Center (Tsukuba, Ibaraki 305-0074, Japan) (RBRC02228). For genotyping, primers “TTGCCGTCTGAATTTGACCTG” and “TCTGCTTCAATCAGCGTGCC” were used to amplify the *LacZ* transgene, and primers “CTAGGCCACAGAATTGAAAGATCT” and “GTAGGTGGAAATTCTAGCATCATCC” were used to amplify the internal control. Wild-type CD1 mice were purchased from the Vital River Company (Vital River Laboratory Animal Center, Beijing, China).

Mice were maintained under specific-pathogen-free (SPF) conditions with a constant photoperiod (12L:12D) and free access to food and water. All mice were bred and used according to the “Guidelines for the Care and Use of Animals in Research” of the Institute of Zoology, Chinese Academy of Sciences, and all experiments which involved the use of the mice were approved by the ethics committee of the Institute of Zoology, Chinese Academy of Sciences (Beijing, China).

### 4.2. Fluorescence Activated Cell Sorting (FACS)

The K14-H2B-GFP/Lef1-RFP mice were euthanatized at indicated postnatal days. Individual mouse dorsal skin was shaved and cut off, after the subcutaneous fat gently removed, the skin was cut into very small pieces and subsequently incubated with 2.5 mg/mL Type IV collagenase (Gibco Laboratories, Carlsbad, CA, USA) in a shaker for 4 h at 37 °C until there was no other solid matter remaining but hair shafts. Single cell suspension was obtained by pipetting, straining, repeated washing, and centrifugation. Three different cell populations (GFP^−^/RFP^+^, GFP^−^/RFP^−^, and GFP^+^/RFP^−^) were sorted from each individual mouse dorsal skin sample in a BD Aria Flow cytometer (Becton-Dickenson, Franklin Lakes, NJ, USA). Sorted cells were then either cytospinned for immunostaining or centrifuged for RNA extraction.

### 4.3. Immunostaining

For Immunofluorescence staining, sorted cells on cover glass or frozen back skin sections were fixed with 4% paraformaldehyde (PFA) (Sigma, St. Louis, MO, USA) for 10 min at room temperature. After washed with PBS for three times, samples were incubated in 5% BSA and 0.2% triton-X 100 (Sigma) in PBS for one hour at room temperature, and then incubated with primary antibodies at 4 °C overnight. For detection, FITC- or TRITC-conjugated secondary antibodies (Zhongshan Goldenbridge Biotechnology Co., Ltd., Beijing, China) were added for one hour at room temperature. After samples were washed with PBS, propidium iodide (PI) or Hoechst 33342 (both form Sigma) was used for nuclear staining. All images were acquired with Nikon Eclipse 80i microscope (Nikon, Tokyo, Japan). The following primary antibodies were used: rat anti-CD45 (1:200, Becton-Dickenson) and rabbit anti-CD117 (1:200, Cell Signaling Technology, Danvers, MA, USA).

### 4.4. Alkaline Phosphatase (AP) Staining

Cells on coverslip were fixed with 4% PFA, then incubated with NBT-BCIP solution (Sigma) for 30 min at room temperature. After washed with PBS, the cells were counterstained with nuclear fast red (Novon Scientific, Beijing, China) and dehydrated with alcohol (Beijing Chemical Works, Beijing, China) gradient followed by xylene (Beijing Chemical Works), then coverslipped with a resinous mounting medium (Shanghai specimen and model factory, Shanghai, China).

### 4.5. R-Spondin1-Fc Protein Production and Verification

Stably-transfected 293T cells producing mouse R-spondin1-Fc fusion protein was a kind gift from Calvin Kuo at Stanford University. This fusion protein contains a C-terminal mouse IgG2-Fc fragment and was purified from the conditioned medium of the R-spondin1-293T cells via protein A affinity chromatography [18]. Briefly, R-spondin1-293T cells were cultured in a Nunc Polystyrene Cell Factory System (Thermo Scientific, Waltham, MA, USA), and the conditioned medium was collected and filtered. The R-spondin1-Fc protein was then purified using protein A Ceramic HyperD^®^ F Affinity Chromatography Sorbent (Pall, Port Washington, NY, USA), and concentrated in saline using the 10 K Amicon-Ultra Centrifugal Filter (Millipore, Billerica, MA, USA). The yield of the purified protein was determined with a BCA Protein Quantification Assay Kit (Beyotime, Nantong, China). The level of R-spondin1-Fc protein was verified by CBB staining and immunoblotting with the R-spondin1 antibody (Santa Cruz Biotechnology, Dallas, TX, USA).

### 4.6. TCF-Luciferase Assay

The TCF-luciferase assay was performed as previously described [18]. Briefly, 293T cells were cultured in DMEM (Gibco Laboratories) with 10% fetal bovine serum (FBS) (Hyclone, Logan, UT, USA), transfected with pTOP Flash or pFOP Flash plasmid together with pRL-TK plasmid using Fugene HD reagent (all from Promega, Madison, WI, USA) for 6 h and then cultured in DMEM with 10% FBS overnight. Cells were starved in serum-free DMEM for 8 h and subsequently treated with different combinations and doses of Wnt3a (Peprotech, Rocky Hill, NJ, USA), R-spondin1 (Sino Biological Inc., Beijing, China), and R-spondin1-Fc fusion protein for 18 h. Luciferase activity was determined using a Dual Luciferase Reporter Assay Kit (Promega) in a BioTek multi-mode plate reader (BioTek Instruments Inc., Winooski, VT, USA) following the manufacturers’ instructions. Relative TCF-luciferase activity was calculated as (TOP-activity/TK-activity)/(FOP-activity/TK-activity). For statistical analysis, each treatment was duplicated, and the data were shown as the means ± SEM.

### 4.7. R-Spondin1-Fc Protein Injection

Telogen CD1 or TOP-Gal mice were injected with R-spondin1-Fc fusion protein according to a schedule shown in Figure 2c. Briefly, R-spondin1-Fc and Noggin (Peprotech) were reconstituted in 0.1% BSA/PBS solution to a final concentration of 100 μg/mL (R-spondin1-Fc) and 5 μg/mL (Noggin). 50 μL solution was intradermally injected into the dorsal skin of a PD56 mouse. This injection was repeated for the next six days. Skin was observed and imaged for the next few days and was harvested before PD80 to identify hair regrowth and precocious anagen entry. For the statistical analysis, considerable hair regrowth at the injection site was regarded as + whereas no regrowth was regarded as −. A chi-squared test was used to evaluate the differences between each two groups (*n* = 5 for the control group; *n* = 6 for the R-spondin1-Fc group; *n* = 4 for the Noggin group).

### 4.8. Skin Harvest and β-Galactosidase (LacZ) Staining

β-galactosidase (LacZ) staining were performed as previously described [36]. Briefly, mouse skin from the mid-dorsal region was harvested and fixed in 4% PFA in PBS for 30 min at 4 °C. The fixed tissues were washed in PBS containing 0.02% NP-40, 0.01% sodium deoxycholate, and 2 mM MgCl_2_ and then incubated overnight in staining buffer (1 mg/mL X-gal, 0.02% NP-40, 0.01% sodium deoxycholate, 5 mM K_3_Fe(CN)_3_, 5 mM K_4_Fe(CN)_6_, 2 mM MgCl_2_, and 0.1 M phosphate buffer, all from Sigma, at 37 °C in the dark. 70% alcohol was used to stop the reaction and store the stained tissue samples. For histological observation, samples were dehydrated through a gradient of ethanol, embedded in paraffin (Leica Instruments, Nussloch, Germany), and then cut into 10–20 μm-thick sections using the CM1950 platform (Leica Instruments). The tissue sections were deparaffinized, rehydrated, and then counterstained with eosin (Zhongshan Goldenbridge Biotechnology Co., Ltd.).

### 4.9. In Vitro Bulge Stem Cell Culture and Treatment

Mouse H2B-GFP bulge cells were a kind gift from Ting Chen’s lab at the National Institute of Biological Sciences, Beijing. Cells were cultured and expanded on NIH-3T3 mouse fibroblast feeder cells treated with mitomycin C (Sigma) as described previously [22]. For stimulation assay, bulge cells were treated with murine Wnt3a (30 ng/mL) and/or human R-spondin1 (100 ng/mL) for 24 h after being starved for 8 h. Bulge cells were then collected for RNA and protein extraction after the feeder cells were removed by trypsinization. Each treatment was triplicated.

### 4.10. MTS Cell Proliferation Assay

Bulge cells were cultured and treated as described above. The cell proliferation assay was performed using CellTiter 96^®^ AQueous One Solution Cell Proliferation Assay (MTS) Kit (Promega) following the manufacturer’s instructions. Absorbance at 490 nm was examined in a BioTek Microplate Reader (BioTek Instruments Inc.). For statistical analysis, every group had a triplicate. Data were normalized to the control group. Data are presented as means ± SEM. and differences were considered significant with a *p* value less than 0.05.

### 4.11. RNA Extraction and PCR

Different cell populations sorted by FACS were centrifuged and the total RNA was extracted using a ReliaPrep™ RNA Cell Miniprep System (Promega). cDNA was generated with a GoScript^®^ Reverse Transcription System (Promega). Semi-quantitative PCR (qPCR) was performed using GoTaq^®^ qPCR Master Mix (SYBR Green) (Promega). The qPCR procedure was 94 °C for 2 min and 45 cycles of 95 °C for 15 s, 55 °C for 15 s, and 68 °C for 25 s. PCR was performed using Promega PCR Master Mix (Promega) and the procedure was 94 °C for 5 min; 24 (only for *Gapdh*) or 35 cycles of 94 °C for 30 s, 55 °C for 30 s, and 72 °C for 30 s; 72 °C for 5 min. The primers used in PCR are listed in Table S1. Relative gene expression versus *Gapdh* expression was calculated using the ΔΔ*C*_t_ method with efficiency correlation [37]. No less than three independent samples were used for statistics and the differences between two groups were analyzed with the independent sample *t*-test. Data are presented as means ± SEM. and differences were considered significant with a *p* value less than 0.05.

### 4.12. Protein Extraction and Western Blotting

The Western blotting protocol was described in detail previously [38]. Briefly, cell extracts were harvested with RIPA lysis buffer (Beyotime) containing phenylmethylsulfonyl fluoride (PMSF) (Beyotime). Then the total proteins were heated at 95 °C for 5 min with 5× SDS loading buffer (Beyotime). The proteins were separated by sodium dodecyl sulfate-polyacrylamide gel electrophoresis (SDS-PAGE) and transferred to polyvinylidene fluoride (PVDF) membranes (Millipore). The PVDF membranes were blocked with non-fat milk (Sigma) and then applied with primary antibodies and horseradish (HRP)-conjugated secondary antibodies (Zhongshan Goldenbridge Biotechnology Co., Ltd.). Membranes were detected with Immobilon Western HRP substrate (Millipore). Quantitative gray-scale analysis was performed using Quantity One software (Bio-Rad, Hercules, CA, USA), using GAPDH as a loading control. Three independent experiments were performed for quantification and the differences between two groups were analyzed with the independent sample *t*-test. Differences were considered significant with a *p* value less than 0.05.

The following primary antibodies were used: mouse anti-active β-catenin (1:500, Millipore 05-665), mouse anti-β-catenin (1:400, Millipore 04-958), rabbit anti-Axin2 (1:2000, abcam ab109307, Cambridge, UK), mouse anti-MYC (1:500, Cell Signaling Technology 3074), rabbit anti-phospho-GSK3β (Ser9) (1:500, Cell Signaling Technology 5558), rabbit anti-GSK3β (1:200, Cell Signaling Technology 9315), and rabbit anti-GAPDH (1:1000, Cell Signaling Technology 2118).

## Supplementary Materials

Supplementary materials can be found at http://www.mdpi.com/1422-0067/17/4/582/s1.
