# Supplementary material for: Exogenous R-Spondin1 Induces Precocious Telogen-to-Anagen Transition in Mouse Hair Follicles
Source: Int J Mol Sci. 2016 Apr 20;17(4):582. doi: 10.3390/ijms17040582 (PMC4849038; doi:10.3390/ijms17040582)
Supplement: Supplementary file 1 [file ijms-17-00582-s001.pdf]

Supplementary Materials: Exogenous R-Spondin1 Induces Precocious Telogen-to-Anagen Transition in Mouse Hair Follicles

Na Li, Shu Liu, Hui-Shan Zhang, Zhi-Li Deng, Hua-Shan Zhao, Qian Zhao, Xiao-Hua Lei, Li-Na Ning, Yu-Jing Cao, Hai-Bin Wang, Shuang Liu and En-kui Duan

Table S1. Primers used for qPCR and RT-PCR assays.

Gene	Forward Primer (5′–3′)	Reverse Primer (5′–3′)
Rspo1	GGGATCAAGGGCAAGAGACAG	CTGGCGGATGTCGTTCCTC
Rspo2	ATAGAGGCCGCTGCTTTG	TGCCGTGTTCTGGTTTCC
Rspo3	TGGATATTACGGAACTCG	CTAACCCTTCTGGGCAAC
Rspo4	CTCGCCCTGTACCGAAGGA	CACTTGCCGTACTGACGGATG
Gapdh	GGTTGTCTCCTGCGACTTCAACAGC	CGAGTTGGGATAGGGCCTCTCTTGC
Ki67	ATCATTGACCGCTCCTTTAGGT	GCTCGCCTTGATGGTTCCT
Ccnd1	GGGATGTGAGGGAAGAGGTGA	GCAGCGAAAACAACGTGAAA
Ccnd2	CTGTGCGCTACCGACTTCAA	CACATCAGTGTGGGTGATCTTG
Ccnd3	TTGCATCTATACGGACCAGGC	AGGAAGTCGTGCGCAATCA
Ccnb1	CTCAGGGTCACTAGGAACACG	AGCTCTTCGCTGACTTTATTACC
Cdkn1a	TCTTGCACTCTGGTGTCT	CTCTTGCAGAAGACCAATCT
Cdkn1b	AATTGGGTCTCAGGCAAAC	CCCTTTTGTTTTGCGAAGAAG
Cdkn1c	ACGAGAATCAAGAGCAGC	AGTTCTCTTGCGCTTGG
Tcf7	GCCAGAAGCAAGGAGTTCAC	ACTGGGCCAGCTCACAGTAT
Cd34	AAGGCTGGGTGAAGACCCTTA	TGAATGGCCGTTTCTGGAAGT
Cd44	AGCCCCTCCTGAAGAAGACT	ACTCGCCCTTCTTGCTGTAG
Sox7	CCCTTACTCACCGGAGTTCA	GGGGACATCCAGAAACAGAG
Sox9	ACTCACATCTCTCCTAATGCT	CGTCGGTTTTGGGAGTG
Sox21	AACACTCGCAGGTGGCG	AGATCGCAGGTCTGCTCTG
Krt10	CGAGGACCTTAAGGGGCAG	CAGGGTCACCTCATTCTCGTAT
Krt17	CAGGCTGGGGTCAGCTAGT	CCAAAGTTGCCTCCATAACC
Krt5	GGAGATCGCCACCTACAGGA	TCCGTAGCCAGAAGAGACACT
Lgr4	CCCGACTTCGCATTCACCAA	GCCTGAGGAAATTCATCCAAGTT
Lgr5	CCTACTCGAAGACTTACCCAGT	GCATTGGGGTGAATGATAGCA
Lgr6	GAGGACGGCATCATGCTGTC	GCTCCGTGAGGTTGTTCATACT
Axin2	AACCTATGCCCGTTTCCTCTA	GAGTGTAAAGACTTGGTCCACC
Myc	GTGTTTCCTGTGTTAATGCCAC	CACAAGTCAGTGCGGTTCAC
Lef1	GCCACCGATGAGATGATCCC	TTGATGTCGGCTAAGTCGCC
Wnt1	CAGGGTTCATAGCGATCCATCT	CCCTCAGGATGGCAAAAGG
Wnt2	GACTACGGGATCAAGTTTGC	CACACCATGACACTTGCA
Wnt2b	CCCTCCACTTCAAGCCTCTGA	AGCCATAGCAGCCCAGGATT
Wnt3	GCCCGCTCAGCTATGAACAA	CCCGTGGCATTTACACTTTAGG
Wnt3a	GCCTCCGCTGGAGTAGCTTT	GGCTGCTGCACCCACAGATA
Wnt4	AGGAGTGCCAATACCAGT	GCTGAAGAGATGGCGTATAC
Wnt5a	GCTTCGCTTGAATTCCTCG	TGGAAGACATGGCACCT
Wnt5b	CCACTGGTGTTGCTTTGTCAGA	CCCGTGTGGTGCAGTCACTT
Wnt6	CCAGCAGCTCCCTAGGAAAAGT	GGCTGGTGTAAACCCCAAGTTC
Wnt7a	CGCCAAGGTCTTCGTGGAT	CGACCCGCCTCGTTATTGT
Wnt7b	CAGCACCAGTTCCGATT	CGTGATGGCATAGGTGAAG
Wnt8a	CCGGCAGATGGGAAATTACC	CTCTGTTGCCAGCCCTTAGCT

Table S1. *Cont.*

Gene	Forward Primer (5′–3′)	Reverse Primer (5′–3′)
Wnt8b	ACCCTGCCTTTCTCCGAAGA	GCGGTGAAGGACACAAGTGACT
Wnt9a	GACAACCTCAAGTACAGCA	ACCATGGCATTTGCAAGT
Wnt9b	GGCCCTTCCTTAAGTCACACAAG	ACAAACAGAGGGTGGCAGAATG
Wnt10a	TCGGAACAAAGTCCCCTACGA	TGCTATGGCGTAGGCGAAAG
Wnt10b	TGCTCTCTTTCAGCCCTTTGC	CTCAGGCAGATGCTCTGAGCTT
Wnt11	AAGGTGGTACACCGGCCTATG	GAGTCCTTCACAGGCCGGATA
Wnt16	GTCATGCAGTGCAGGCAAC	GCCTTCACTTGGTGAGCCAC
Alx4	GGACGGTAGCCTCAAGCTC	CACGGGTTCAGAGTCAGGA
Fgf7	ACCTGAGGATTGACAAACGAGG	CCACGGTCCTGATTTCCATGA
Vim	CGGCTGCGAGAGAAATTGC	CCACTTTCCGTTCAAGGTCAAG
Krt14	AGCGGCAAGAGTGAGATTTCT	CCTCCAGGTTATTCTCCAGGG
Kit	TGACGGTACATGGCTGCATT	ACCACCGTAAATGTGTCCCC
Ptprc	GTTTTCGCTACATGACTGCACA	AGGTTGTCCAACTGACATCTTTC
Gsk-3β	TGGTGTGGATCAGTTGGTGG	GGCCGGAAGACCTTTGTCC
β-catenin	ATGGAGCCGGACAGAAAAGC	CTTGCCACTCAGGGAAGGA

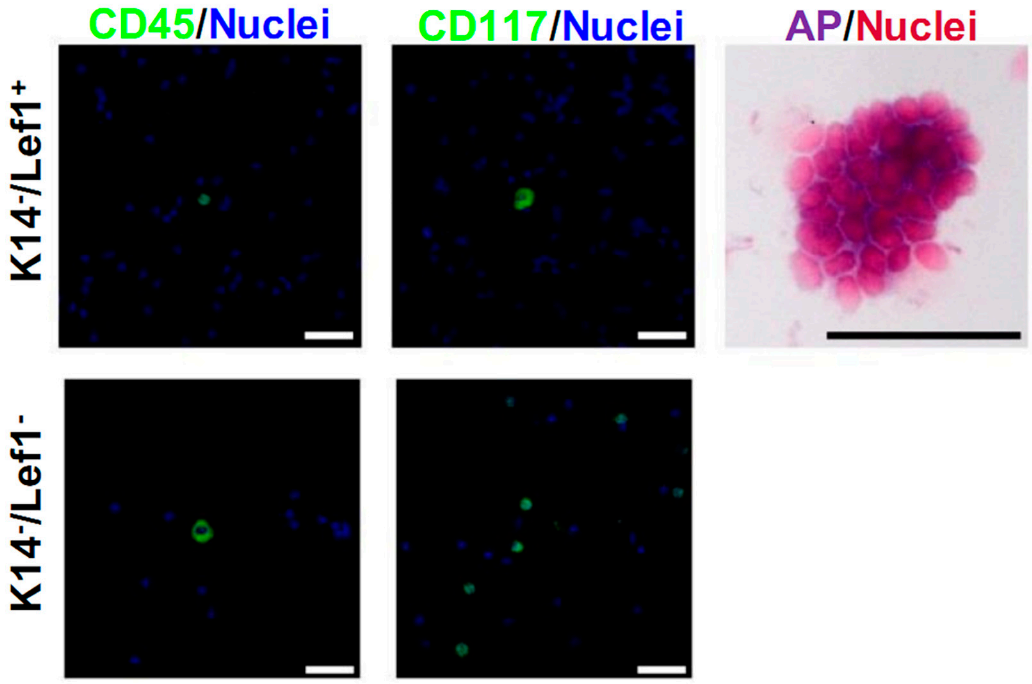

Figure S1. Immunostaining of CD45 and CD117 in the K14⁻/Lef1⁺ and K14⁻/Lef1⁻ cell populations and AP staining in K14⁻/Lef1⁺ cells. AP, alkaline phosphatase. Bar = 50 μm.

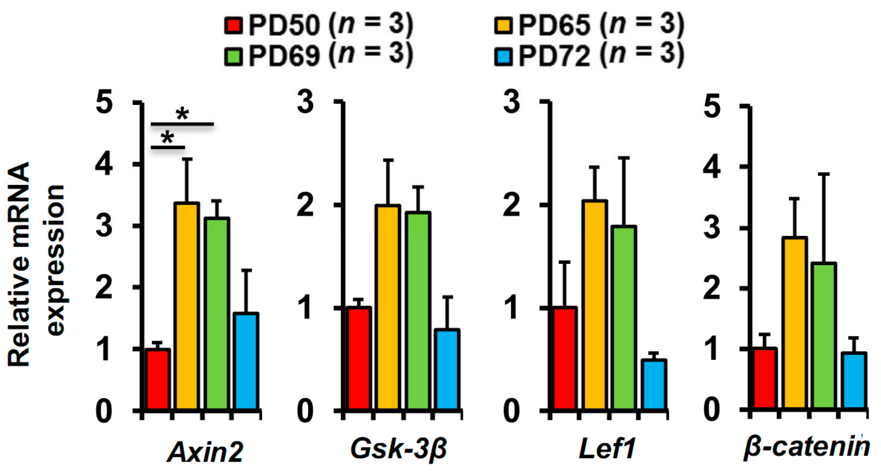

Figure S2. Relative expression of Wnt signaling related genes in the DP in different periods of telogen. PD, postnatal day. * $p < 0.05$.

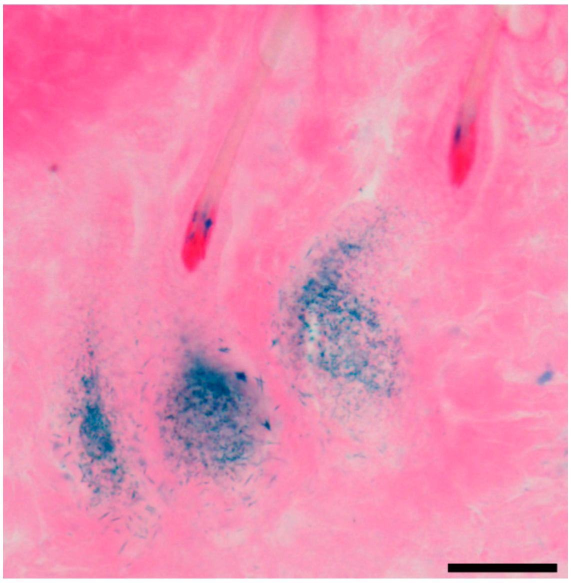

Figure S3. X-gal staining of dorsal skin of TOP-Gal mice injected with R-spondin1-Fc one week after the initial injection with eosin counterstaining. Bar = 50 μm.

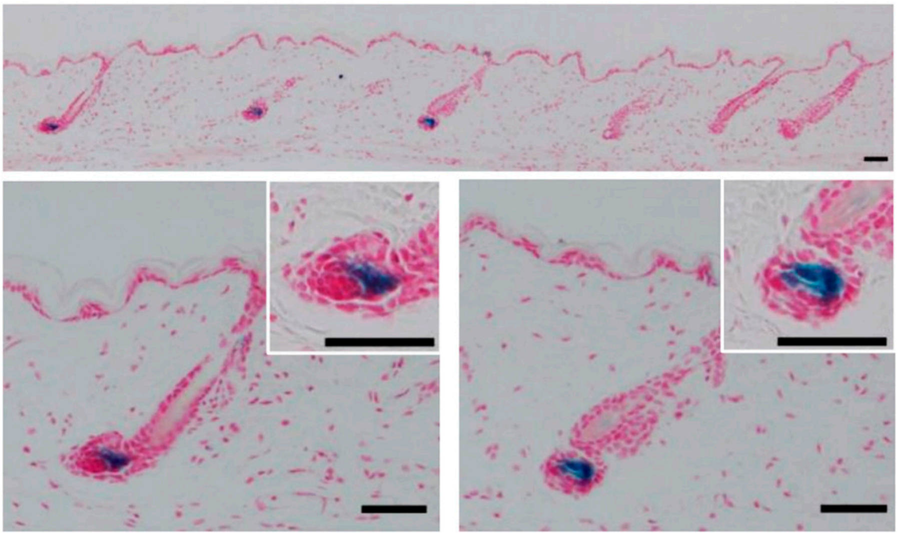

Figure S4. X-gal staining of dorsal skin of TOP-Gal mice injected with BSA on PD76 with nuclear fast red counterstaining. Bar = 50 μm.

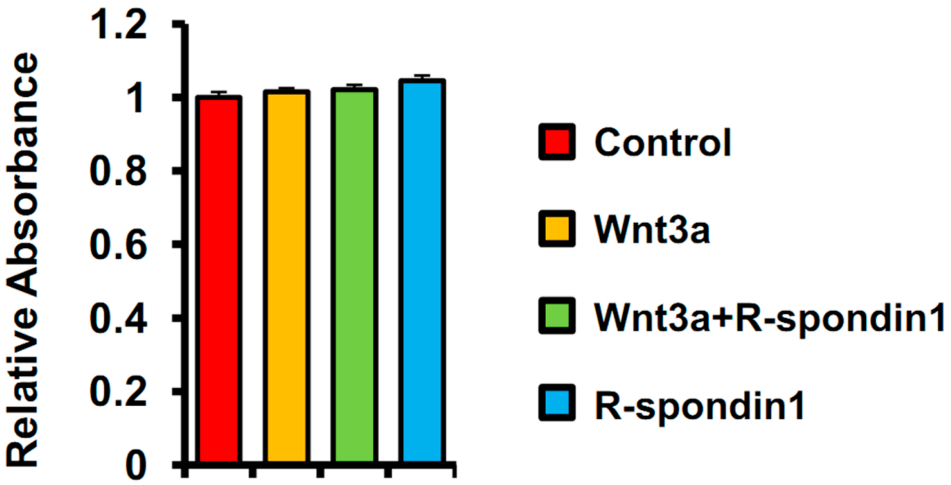

Figure S5. MTS assay of bulge stem cells treated with Wnt3a or/and R-spondin1 for 24 h following an 8 h starvation.

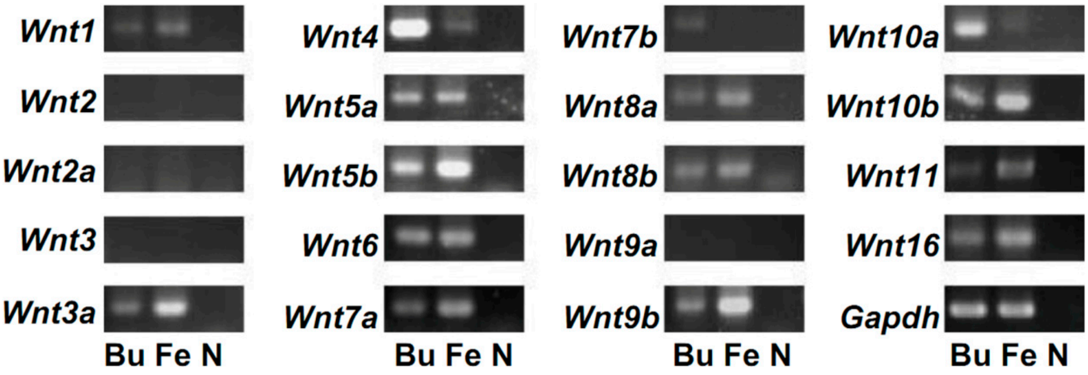

Figure S6. RT-PCR showing the expression of different Wnt ligands in bulge stem cells (Bu) and NIH-3T3 feeder cells (Fe). H_2O was used instead of cDNA as a negative control (N) and *Gapdh* expression was assessed as an internal control.
